# *“Every drug goes to treat its own disease…”* – a qualitative study of perceptions and experiences of taking anti-retrovirals concomitantly with anti-malarials among those affected by HIV and malaria in Tanzania

**DOI:** 10.1186/1475-2875-13-491

**Published:** 2014-12-13

**Authors:** Peter E Mangesho, Joanna Reynolds, Martha Lemnge, Lasse S Vestergaard, Clare IR Chandler

**Affiliations:** National Institute for Medical Research, Amani Medical Research Centre, P.O. Box 81, Muheza, Tanzania; Department of Social and Environmental Health, London School of Hygiene & Tropical Medicine, 15-17 Tavistock Place, London, WC1H 9SH UK; National Institute for Medical Research, Tanga Medical Research Centre, P.O. Box 5004, Bombo Road, Tanga, Tanzania; Centre for Medical Parasitology at Department of International Health, Immunology and Microbiology, University of Copenhagen and Department of Infectious Diseases, Copenhagen University Hospital (Rigshospitalet), Øster Farimagsgade 5, Bygning 22, 1014 Copenhagen K, Denmark; Department of Global Health & Development, London School of Hygiene & Tropical Medicine, 15-17 Tavistock Place, London, WC1H 9SH UK

**Keywords:** HIV, Malaria, Concomitant conditions, Tanzania, Qualitative research

## Abstract

**Background:**

Little is known about how people living with human immunodeficiency virus (HIV) experience malaria and the concomitant use of anti-malarial treatments with anti-retrovirals (ARVs). An understanding of how patients make sense of these experiences is important to consider in planning and supporting the clinical management and treatment for co-infected individuals.

**Methods:**

A qualitative study was conducted in Tanzania alongside a clinical trial of concomitant treatment for HIV and malaria co-infection. Focus group discussions were held with people receiving treatment for HIV and/or malaria, and in-depth interviews with health workers responsible for HIV care and members of the clinical trial team. Data were analysed inductively to identify themes and develop theoretical narratives.

**Results:**

Results suggest that people living with HIV perceived malaria to be more harmful to them due to their compromised immune status but saw the disease as unavoidable. For those enrolled in the clinical controlled study, taking anti-malarials together with ARVs was largely seen as unproblematic, with health workers’ advice and endorsement of concomitant drug taking influential in reported adherence. However, perceptions of drug strength appeared to compel some people not enrolled in the clinical study to take the drugs at separate times to avoid anticipated harm to the body.

**Conclusions:**

Management of HIV and malaria concurrently often requires individuals to cross the domains of different disease programmes. In the context of a trial concerned with both diseases, patients experienced the support of clinicians in guiding and reassuring them about when and how to take drugs concomitantly. This points towards the need to continue to strive for integrated care for patients with HIV.

## Background

The global burdens of malaria and human immunodeficiency virus (HIV) are high, together causing over 2.5 million deaths in 2009 [1, 2]. In many sub-Saharan African countries, including Tanzania, populations are at risk of HIV and malaria concurrently. However, action to tackle the two diseases is often carried out in parallel. The need for coordinated and integrated approaches is recognized as essential, and a focus has been placed on health systems as a whole in supporting programmes for different diseases [3, 4]. The negative clinical consequences of HIV-malaria co-infection are already well established, including a higher risk of malaria and developing more severe clinical symptoms amongst HIV-positive people, as reviewed by C Flateau *et al*. [5]. Investment in supply of effective anti-malarials and anti-retrovirals (ARVs) has been significant [6], and there is also an increasing knowledge about the potential therapeutic implications of treating co-infected individuals. Thus recent studies suggest that drug-drug interactions between certain anti-malarials and anti-retrovials exist, which affects achieved drug concentrations and hence may influence the efficacy and/or the safety profiles of those treatments, depending on the medicine combination [7–9].

Despite the relationship between the two diseases, however, the management of treatment for malaria and HIV co-infection has received comparatively little attention, and there is a paucity of research around the experiences of people needing receiving treatment for co-infection, and how patients manage concurrent medicine taking. Beyond the clinical implications of treating co-infection of HIV and malaria, greater understanding of people’s experiences of taking concomitant treatments is imperative to support the management of these diseases together. A wide literature on ‘adherence’ to treatment regimens for various diseases has highlighted the importance of attending to experiential accounts of taking medication, to understand the range of social, physical and material factors which can shape patients’ treatment-taking and seeking behaviours [10, 11]. There are clear clinical implications of patients not seeking or following treatments as recommended either for malaria [12] or HIV [13]. In addition, the management of treatment at the individual level carries implications for broader public health, specifically in terms of the potential impact on the development of resistance to malaria or HIV drugs [14, 15]. These issues are likely to be compounded by patients’ experiences of taking HIV and malaria treatments concomitantly for co-infection.

The meanings of malaria and of HIV and AIDS to those affected across the African continent have been explored extensively, leading to two large, but mostly separate, bodies of literature [16, 17]. These works have broadly examined how conceptualizations and experiences of disease, treatment options, and perceptions of effectiveness and side effects of medication may influence treatment-seeking and prevention behaviours for either HIV or malaria alone [18–22]. There is a notable absence of literature about people’s experiences of treatment and taking medications concomitantly for HIV and malaria co-infection. Studies have explored experiences of concomitant treatment for HIV and other co-infections, such as tuberculosis (TB) or hepatitis C [23–25], although this remains largely in the domain of clinical or pharmacokinetic research [26].

A qualitative study was designed to understand how people who straddle these two diseases of HIV and malaria make sense of their situation and especially the concomitant drugs to be taken. The study drew on theoretical perspectives from medical anthropology, particularly considering the ‘charm’ or ‘power’ of medicines [27].

## Methods

### Study setting

This research took place at Muheza District Hospital in northeast Tanzania where malaria is endemic, and prevalence of HIV is estimated at 6.7% [28]. The hospital had a separately funded and slightly separated HIV centre where all HIV positive patients could receive ongoing care for HIV and other illnesses. Treatment for malaria for HIV-positive patients is supposed to be free although medicines were not always available and patients at times had to buy them from other public or private facilities. The current nationally recommended first-line treatment for malaria in Tanzania is artemether-lumefantrine (ALu), and for HIV a selection of nevirapine- or efavirenz-based combinations.

The qualitative study was conducted in 2011 alongside a clinical controlled study (trial number NCT00885287, [29]) which assessed the therapeutic efficacy, clinical safety and pharmacokinetic interactions of anti-malarials taken concomitantly with anti-retrovirals in adults co-infected with HIV and uncomplicated malaria, against comparator groups of HIV-positive adults co-infected with malaria but not taking anti-retrovirals, and HIV-negative adults with malaria between 2009 and 2012. Participants for the clinical study were recruited among patients presenting with fever at the HIV centre or general outpatients section of the hospital, where they were tested for malaria using rapid diagnostic tests (RDTs). If testing positive for malaria they were invited to join the clinical study, received free treatment for malaria and were monitored with frequent follow-up visits and repeated malaria testing over 42 days.

### Qualitative study design

A qualitative methodology based on an interpretative (meaning-based) approach [30] was employed to explore people’s perceptions and experiences of taking medicines for HIV and malaria. Focus group discussions (FGDs) were selected to provide an opportunity to explore experiences and points of reference that are shared and those in dispute within specified sub-groups, through observing social interactions in groups [31]. In-depth interviews (IDIs) were considered appropriate for understanding the perspectives of individuals and gaining narratives of their experiences with particular topics [32].

### Sample

To explore a range of perspectives on taking HIV and malaria treatment concomitantly the study sampling strategy was devised around three sub-groups of ‘patients’ who had received treatment for HIV and/or malaria, and an additional sub-group of health workers who had experience of delivering care to HIV patients either within or beyond the clinical study. The three sub-groups of treatment recipients included: a primary sub-group (A) of people who were HIV-positive and taking ARVs, and who had recently been diagnosed with malaria and treated with ALu as part of the clinical study; a comparator sub-group (B) of HIV-negative people who were diagnosed with malaria and treated with ALu and had participated in the comparison arm of the clinical study (group B); and a second comparator sub-group (C) of HIV-positive people taking ARVs who had not been eligible to receive ALu as part of the clinical trial, either because they were not diagnosed with malaria or had taken other anti-malarials already (group C). To recruit participants for the treatment recipient sub-groups A and B, the clinical study database was used, working backwards in time from those who had most recently completed the 42-day follow-up period, to limit the effects of difficulty with recall over time. Participants for sub-group C were identified prospectively as part of the ongoing screening and recruitment for the main clinical study. The health worker sub-group reflected a purposive and convenience sample of clinicians and nurses working on the clinical study, health workers delivering care at the hospital HIV clinic and other hospital staff from the in- and out-patients departments.

The planned sample sizes were three to four focus group discussions per ‘patient’ sub-group, with between eight and 12 participants in each, and eight to ten individual in-depth interviews with health workers. These sizes reflected the intentions of the study to explore a range of experiences and to be able to make comparisons between different sets of perspectives, while acknowledging the scope and resource limitations posed by in-depth qualitative research methods.

### Data collection

FGDs with the three groups of treatment recipients explored perceptions and experiences of taking ARVs with anti-malarials as well as other medicines. FGDs were separated by HIV status and gender to minimize concerns around disclosure of HIV status and to facilitate open discussion. IDIs with health workers explored attitudes and experiences of providing care to patients co-infected with HIV and malaria. Semi-structured topic guides were developed and piloted for both the FGDs and IDIs.

PM facilitated the group discussions and interviews in Kiswahili, English, or a mixture. With participants’ permission, FGDs and IDIs were audio-recorded and a note taker was present in the FGDs to record both verbal and non-verbal communications. FGDs took place in a room away from the main hospital site, and IDIs were conducted in a private space on the hospital site. All recordings were transcribed verbatim in Kiswahili, translated into English and cross-checked for accuracy.

### Data analysis

All transcripts were organized and coded by PM and JR with the assistance of QSR Nvivo 8 qualitative data management software [33]. The coding process was informed by principles of grounded theory [34] whereby analytical categories and concepts were identified by going through each transcript line-by-line, identifying potential themes, in an iterative, comparative process. Interpretations were further developed through discussion among the research team.

### Ethical considerations

The study was approved by the ethical committee of the London School of Hygiene & Tropical Medicine (reference A216 5828) and the National Institute for Medical Research in Tanzania (reference NIMRlHQ/R.8alVol. IX/1150). The FGDs were segregated by HIV status, but the nature of the discussions meant that HIV-positive participants’ status was likely to become known to other participants during the FGDs. Participants were made aware of this at the point of invitation to participate, and reminded at the beginning of the discussion not to share the content of the discussion beyond the group. After providing information about the purpose and procedures of the study, written consent to participation and being audio-recorded was taken from each participant. For participants unable to write, a thumb-print was accepted in place of a signature.

## Results

### Participants

A total of 13 FGDs were conducted. On average there were eight to ten participants per discussion with a total of 114 participants (see Table 1). More women participated in the FGDs than men (76 and 38 respectively), which reflected a greater number of women presenting to the clinic for HIV treatment. Amongst those with malaria but not HIV, equal numbers of each gender participated. In total, ten health worker IDIs were conducted. There were no refusals to participate. The results presented here focus primarily on the experiences and perspectives of those participants living with HIV, and distinctions with the other study group participants are highlighted where pertinent.

**Table 1:** **Number of FGDs and IDIs conducted by sub-group**

Group	Sub-group	Number completed	
		Male	Female
**FGDs**			
Treatment recipients	A (HIV-positive, receiving ARVs, completed participation in clinical controlled study of Alu efficacy and safety	1	3
	B (HIV-negative, no ARV, completed participation in clinical controlled study of ALu efficacy and safety)	2	2
	C (HIV-positive, receiving ARVs, excluded from participation in clinical study)	1	4
**IDIs**			
Health workers	Clinical study staff	4	
	HIV Care and Treatment Clinic health workers	4	
	Hospital health workers	2	

### Paradigms of ARVs and anti-malarial medicines

For those living with HIV, general use of medicines was made sense of in the context of their different experiences of sickness and treatment which were mediated by the different programmes or health services on offer. When taking ARVs and attending the HIV centre regularly, people had been taught about adherence to treatment and the need for this was framed in stark terms. The role of ARVs was perceived as to prolong life: *“You take ARVs so that you live longer, like food, if you do not eat you die*” (Respondent (R) 02; FGD A2, HIV-positive male). Taking ARVs was described as a ‘contract’ – ‘*mkataba’* – between patients and the drugs, and therefore implied certain rules around taking drugs, which had to be adhered to. In particular, the notion of timing of drug-taking was prominent in participants’ narratives. Some described choosing their own schedule whereas others indicated the timing was directed by a doctor. Adherence to the daily schedule of taking ARVs was indicated as important for their effectiveness and to prevent harm:

*… you cannot be harmed by the drug if you concentrate on the schedule of the drug taking* (R 03; FGD C3, HIV-positive female).

The framing of taking ARVs in terms of timing continue into some accounts of taking ARVs concomitantly with other drugs, as described in more detail below.

Other medicines, including anti-malarials, were often spoken about in rather different terms, outside of the HIV treatment paradigm. Such drugs could be purchased or obtained easily, with minimal discussion with the vendor or healthcare provider. Malaria, especially, was considered a ‘normal’ disease for which a range of possibilities of treatment were presented. The first-line anti-malarial drugs, artemether-lumefantrine, were the outcome of a linear, biomedical process of seeing the doctor, receiving a diagnosis (including getting tested), prescription of medicine and getting cured. This mirrored the way anti-malarials were discussed in the HIV-negative focus groups. Being tested prior to receiving medicines was articulated in the FGDs with HIV-positive people as very important in the fulfilment of this treatment process, for example attending a health facility to be tested for malaria, if experiencing malarial symptoms. While some participants mentioned traditional medicine and miracle healing, most respondents intimated that these were not as effective as biomedical treatment, though this could reflect participants’ reluctance to disclose what might be considered ‘deviant behaviour’ in the context of our study based within the hospital and a clinical research study. Effectiveness of a treatment was perceived to be linked to eradication of symptoms or proof from a medical test that the disease was eradicated.

### Making sense of concomitant medicine taking

Given the two different paradigms of medicine taking, one mediated through the HIV treatment programme of clearly defined protocols, and the other developed through long histories of familiarity with malaria as a disease and easy availability of treatment, HIV patients had to make sense of concomitant medicine taking by straddling these two paradigms. Some made sense of this by extending the ideas developed within HIV treatment programmes to follow the protocol set out, respecting biomedical authority, in the form of doctors’ instructions, over the taking of medicines. However, in the realities of accessing and taking the drugs, challenges emerged relating to diagnosis, pill burden and concepts of medicine strength. Some respondents’ experiential accounts of how they managed mixing medicines in their daily lives suggested adaptations to formal protocols may emerge due to both practicalities and conceptualizations of taking the drugs together.

### Respecting biomedical protocols

Reflecting the discrete approach to HIV and malaria as enacted through disease control programmes, many respondents spoke of their drugs as working independently in the body to address separate health problems: “e*very drug one takes goes to treat its own disease in the body”* (R 07; FGD A3; HIV-positive male). Acquiescing to the authority of biomedicine as experienced through ARV treatment programmes, many patients followed the lead of biomedical workers in their responses to concomitant medicine taking: “*If the doctor prescribes you should take all the medicines together, you have to do it and that is what I do”* (R02; FGD-A4; HIV-positive female). With this perspective, mixing anti-malarials with ARVs was conceptually seen as unproblematic, provided that certain conditions were adhered to, with each treatment perceived as being effective in its own way. These conditions reflected the instructions given by health workers at the clinics, and included eating properly, not taking medicines without prior testing such as confirming malaria (slide or approved rapid diagnostic tests) and taking appropriate doses for one’s weight. This was echoed by some health workers who described HIV-positive patients’ concerns about taking drugs for other infections without direction from doctors:

*… they hesitate until they are advised by the doctor, [the patient] can’t just fall sick and purchase drugs from the shop, most of the time when they come for the doctor’s consultation, that’s when they are told ‘you can use this’ or ‘you cannot use this’.* (IDI 08, hospital doctor).

Further, the stance on proper testing was emphasized by those who believed mixing ARVs with other drugs may cause some problems if proper medical tests and weights described above, were not performed, such as simply purchasing drugs from shops:

*Negative effects to someone can occur if one purchases drugs from the shop without performing proper necessary tests. The drugs can affect you because you do not know the dose you are given from the shop is correct or not, yet also you are on ARVs, so when taking them and they go and badly interact and bring problems because you have not done any tests and received assurance from the doctor on how to use the drugs. (R 08;FGD-C1; HIV- positive Female)*

### Realities of access to drugs

However, this view of the importance of attending a formal health facility to be tested if experiencing malaria-like symptoms before receiving appropriate treatment was tempered by reports of confusion by participants who had experienced malarial symptoms but subsequently tested negative for the disease. A few people suggested this might be linked to the nature of the ARVs they were taking, which could cause similar symptoms to malaria: “.*..sometimes the drugs’ condition can change… these drugs sometimes bring tiredness*” (R03; FGD A1; HIV-positive female). This situation posed a challenge to participants seeking relief for their symptoms, but who sought to adhere to their perceived responsibility to take medicines only after testing.

The view of what ‘should’ be done in the case of malaria-like illness was also contrasted by discussions of self-medication and seeking informal health services, which were woven through accounts of treatment-seeking among both HIV-positive and HIV-negative participants. Such practices are the norm for malaria, and were underpinned by a number of factors, including distance to the health facility, lack of money for transport and fear of stigma for HIV-positive patients. In all these situations, poverty was reported to be a major cause compelling people to self-medicate.

### Realities of pill burden

Taking ARVs concomitantly with anti-malarials was spoken about by some in terms of the number of pills, and the challenges faced in taking these. Most respondents articulated the necessity of taking the numerous pills in order to get better, provided that they had been directed by a doctor. A few stated that without such instructions, taking many pills could be poisonous to the body. This reflection of a biomedical discourse was also apparent in descriptions by several participants of the additional medicines they took for conditions over and above HIV and malaria, for example TB or high blood pressure, and how they were ‘used’ to taking numerous drugs. However, there were also accounts of the efforts involved in persevering with regimes, and descriptions of the support needed by some patients – including those who had not participated in the clinical study – to achieve the ‘successful’ taking of all the necessary medicines:

*… at that moment I was taking nine tablets a day….it reached a point I wanted to stop taking them… And if I was alone until now I would not have felt better, [but] I thank my children and my relatives, they encouraged me.* (R 02; FGD C2; HIV-positive female)

Health workers were vocal in expressing their concern over pill burden to patients, arising from contact with patients when prescribing medicines. They reported that patients expressed fear when prescribed what they [patients] thought to be many pills, *“Patients sometimes are worried about the anti-malarial dose quantity especially if they are also taking ARVs’* (IDI 08, Hospital-Doctor). As a result, some health workers articulated their perceived duty to provide assurance that the pills would not be problematic. This challenge was particularly associated by health workers with patients who had recently commenced ARVs and the problem was said to be complex for patients taking their pills at home with health workers fearing that they did not complete their doses.

### Timing drugs

The notion of timing was also identified as important for taking drugs concomitantly, with some participants highlighting health workers’ role in stipulating the schedule for drug-taking. As such, this echoed a formal biomedical discourse in general, and ARV treatment programme protocols more specifically, whereby medical authority over drug-taking was respected by patients, and appeared – for some participants at least – to be accepted:

*Facilitator (F): When you take [the drugs] at the same time, what is the problem?*

*Several respondents: There is no problem.*

*R 06: Because you are following the doctor’s advice.” (FGD A4; HIV-positive females).*

Perceived medical authority also seemed to play a role in some participants’ acceptance of the scheduled timing of taking ARVs and anti-malarials, be it at the same time or separately. Provided doctors had *“arrange[d] themselves when to take the drugs*” (R 04; FGD A3; HIV-positive male), these participants did not consider there to be a problem with taking treatments concomitantly.

However, a contrasting set of responses, identified predominantly in the FGDs with people who had *not* participated in the clinical study (sub-group C), conveyed a sense of caution towards mixing drugs that was not alleviated by the reassurances of health workers – perhaps because of the non-trial setting. Participants across the study sample perceived that the competing strength, or ‘*nguvu’*, of medicines such as anti-malarials and ARVs could lead to ‘*friction*’ or bad interactions. Several participants described this in relation to perceptions of their own health and strength, indicating that possible negative effects of taking drugs at the same time were linked to their individual condition, in some cases suggesting they adjusted the schedules of drugs to accommodate their perceived strength in their own body:

***F****: What time is bad or good for taking drugs at once?*

***R 02:****You can use malaria drugs and ARV and this depends on your strength. For me, I can’t take [them] at once, I have to have intervals [between them].* (FGD C2, HIV-positive male).

*It is important to separate the immediate combination of the strength from ARVs and the other drug. Because every drug has its own strength. I may have to wait for a certain time, like one hour and a half, the first drug taken would have already be absorbed in its place inside the body.* (R04; FGD-C1; HIV-positive Female)

This account of drug strength and importance of separating them also arose within a group of those who participated in the trial. In this group two conflicting perceptions were reported:

*If I take ARVs at 6 am and I also have to take those of malaria I extend like to wait two up to three hours. This is because even the doctor also told me some drugs can harm you if you take concomitantly so you have to separate time. (R 09; FGD-A3- HIV positive Male)*

*I take them at the same time because every drug does its work at its section for example there was a time I had to take 20 tablets and I take all because each works differently so I think there is no problem with it.* (R 07; *FGD-A3- HIV positive Male)*

There were also several accounts of strategies adopted by people to separate the timing of taking ARVs and other drugs to limit the perceived anticipated negative impact on their daily lives. Sometimes speaking about medicines in general, rather than anti-malarials specifically, several participants cited experiencing ‘*body fatigue’* and ‘*exhaustion’* as a result of taking drugs at the same time. They described the importance of separating the timing of drug-taking to ensure they were able to continue with their work and other daily, household survival strategies:

… [*if you do not separate the drugs] after taking the drugs you [need to] find a place and rest… If you haven’t finished your activities, it means you’ll hurt yourself, working while the drugs are also working in your body.* (R 08; FGD C3; HIV-positive female).

## Discussion

This study was conducted to begin to address the paucity of literature on how people respond to HIV and malaria co-infection, two major diseases affecting sub-Saharan Africans, through specific focus on how people make sense of concomitant treatments. Results from this qualitative study show how people with HIV as well as malaria have to straddle two paradigms of treatment – the first as participants of a protocol of care under the attention of dedicated specialists, and the second as active agents seeking care for a familiar disease for which treatment can be found with minimal biomedical expertise required. Making sense of the cross-over of these paradigms through experiences of co-infection, participants of the trial who received careful advice on taking medicines reiterated the acquiescence to biomedical authority also seen in ARV programmes. However, some, including those who had been excluded from the trial, drew on broader experience with self-medication of malaria to consider circumventing this authority in treatment-seeking as well as tinkering with timing of drugs. This potential for deviations attended to both conceptual understandings and practicalities of concomitant medicine taking, and requires further research.

In the context of the clinical trial where participants were provided with comprehensive care and advice, as well as small facilitators such as milk for swallowing medicines, many appreciated and accepted the protocols of concomitant medicine taking. This mirrored their acceptance of ARV regimens within treatment programmes, and was reflected in the articulated notion of the ‘lifelong contract’ of receiving ARVs and, in turn, in the general acceptability of mixing drugs when regimens had been ‘authorized’ by doctors. In this setting in Tanzania, this may reflect HIV-positive people’s sense of responsibility towards adherence to treatment regimens, produced through hierarchical relations between health workers and patients [35] which have become institutionalised in HIV disease control programmes.

This acceptance of the protocols of treatment, particularly for HIV, stood in contrast to discussions of routine treatment of malaria, which involved more personal experience of self-medication including choice of drugs as well as timings, relating to experiences of the strength of different drugs in one’s own body. While the harmful effect of HIV co-infection on malaria incidence and disease severity is now well documented in the academic literature, including more frequent and more severe malaria illness episodes [36], we found that people living with HIV saw malaria as unavoidable, even ‘normal’. This echoes the narratives of acceptance of misfortune which can be found in numerous contexts where resources are scarce and choices are limited [37].

When it came to managing concomitant medicines, the realities of accessing treatment, the number of pills to be taken and their perceived strength meant challenges for many. While some reported adhering to protocols, others spoke of adjusting the timing of taking different drugs in order to accommodate perceptions of potential ‘friction’ between strong drugs, or to accommodate life in general. Given the special sample of participants involved in this study, associated with a clinical trial, these themes require exploration in further research that follows the everyday lives of people living with HIV as they navigate the needs of concomitant medicines. Such research should also investigate further the requirements of patients for stepping across these different paradigms of different disease control programmes. These early findings support the call for health services to be oriented around patient needs rather than the needs of specific disease control programmes [38] and supports continued efforts towards ‘integrated care’.

This research, where acceptance of protocols was rooted in the relationship between patients with health workers providing a good service in a trial, and the authority embedded in HIV treatment programmes, suggests that promoting safe treatment practices of concomitant medicines that are routinely taken as ‘normal’ such as anti-malarials may be challenging. Public health interventions have tended to focus on health promotion through mass communication efforts, informing population groups of lifestyle risks and preventive behaviours that will seal their fates as ‘ill’ or ‘healthy’ [39]. The premise of such interventions is that individuals can make choices, and that the threat of specific forms of ill-health tomorrow will outweigh other concerns and change behaviour today. Much research now suggests that such interventions are often unsuccessful because assumptions of individual agency do not hold: political and economic circumstances often constrain individual choices [40, 41]. Furthermore, social commitments are often given greater weight in daily decisions than individual health-related activities [42]. In Tanzania, malaria-related risks are played out against local livelihood activities, such as farming, and particular social engagements including funerals, which can shape sleeping patterns and access to nets on a day-by-day basis [43]. In line with findings among pregnant women in Malawi [44], the lived realities of the study participants suggests that for people living with HIV, the dangers of malaria are known, and the authority of biomedical care is recognized, but that test-before-treatment models of dealing with ill health were impractical or irrelevant in the context of their lives. The adherence to protocols reported by many in this study therefore signals that other interventions are required, and that bringing together care for patients under one paradigm may be key to success.

### Limitations

Although a range of perspectives were sought in this qualitative study, the majority of participants included may have different experiences and perceptions from other people facing HIV and malaria co-infection, due to their previous participation in the clinical study. Despite being observational in design, offering the same treatments as routine care in the surrounding health service, the clinical study led its participants to conceptualize it as offering an enhanced ‘service’ [45]. The elements appearing to comprising this perceived service included engaging with the study team, materials and processes over an extended period of time, as well as additional benefits such as transport fare, which were interpreted as expressions of individualized support from the study team. The potential uniqueness of the sample of HIV-positive people who had participated in the clinical study is a limitation for generalizing the study findings more broadly, particularly given the differences interpreted between people who did and did not participate. However, there is also potential to learn from the social and material dynamics underpinning experiences of being in a clinical study for informing routine care and how HIV-positive people can be supported to align the challenges faced in their daily socio-economic lives with the requirements of treatment regimes [35, 46].

## Conclusions

Management of HIV and malaria concurrently often requires individuals to cross the domains of different disease programmes. In the context of a trial concerned with both HIV and malaria, patients experienced the support of clinicians in guiding and reassuring them about when and how to take drugs concomitantly. This presented an ‘integrated care’ scenario which appeared to support patient confidence in concomitant medicine taking following biomedical protocols. This points towards the need to continue to strive for integrated care for patients with HIV.

## Abbreviations

ACT: Artemisinin-based combination therapy
ALu: Artemether-lumefantrine
ARVs: Antiretrovirals
FGD: Focus group discussion
HIV: Human immunodeficiency virus
IDI: In-depth interview
TB: Tuberculosis.
